# Case 6/2017 - A 28-Year-Old Man with Anasarca And Restrictive Heart Disease

**DOI:** 10.5935/abc.20170184

**Published:** 2017-12

**Authors:** Desiderio Favarato, Luis Alberto Benvenuti

**Affiliations:** Instituto do Coração (InCor) HC-FMUSP, São Paulo, SP - Brazil

**Keywords:** Heart Failure, Cardiomyopathy, Restrictive, Pleural Diseases, Heart Diseases, Pleural Effusion, Edema

The patient was a 28-year-old male, born and living in the city of São Paulo, who sought medical care complaining of dyspnea and edema.

He reported abdominal pain and abdominal volume increase five years before, when he sought medical care. On the occasion a gastroenterological investigation began, and a biopsy was suggested, but refused by the patient, who remained without an etiological diagnosis. One year later, edema of the lower limbs appeared, followed by dyspnea on intermediate exertion two years later. The patient reported worsening of the abdominal volume increase, edema of the lower limbs and dyspnea even at rest in the past two months. He was then referred to our hospital.

His physical examination (Aug 11, 2005) showed a regular general state of health, heart rate of 120 bpm, and blood pressure of 90/60 mm Hg. His lung auscultation showed rales up to the middle third of the left hemithorax. His heart auscultation showed arrhythmic cardiac sounds and systolic murmur in mitral area and left sternal border. The liver was palpated 4 cm from the right costal margin. There were ascites and edema of the lower limbs.

His electrocardiogram (Aug 11, 2005) showed atrial fibrillation, low-voltage QRS complexes, parallel SÂQRS + 90º (Figure 1).

**Figure 1:**
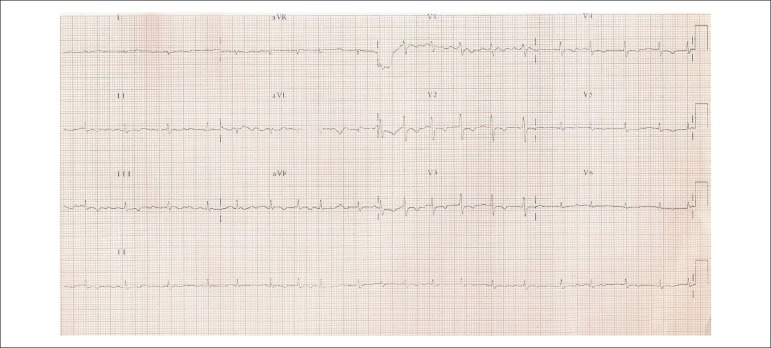
Atrial fibrillation, low-voltage QRS complexes

Decompensated heart failure and left pleural effusion were diagnosed, and the patient was hospitalized for treatment.

His laboratory tests (Aug 13, 2005) revealed: hemoglobin, 14.7 g/dL; hematocrit, 44%; leukocytes, 6400/mm^3^; platelets, 210,000/mm^3^; urea, 30 mg/dL; creatinine, 1.1 mg/dL; potassium, 5.6 mEq/L; sodium, 134 mEq/L; INR, 2.2; TTPA(rel), 1.3; and normal urinalysis.

His serologies for Chagas disease, hepatitis, HIV and syphilis were negative. His antinuclear antibody and rheumatoid factor tests were negative, as were the tests for the following antibodies: anti-smooth muscle (kidney and stomach), anti-mitochondrial, anti-parietal cell, anti-liver cytosol, anti-microsomal fraction, liver/kidney microsomal. Serum copper was normal, and ceruloplasmin was high.

His laboratory tests revealed: brain natriuretic peptide (BNP), 192 pg/mL; TSH, 17.7 µU/mL; T4L, 1.1 µg/dL; ferritin, 202 µg/dL; serum iron, 36 µg/dL; iron saturation, 25%; factor V activity, 36%.

His chest computed tomography (Aug 15, 2005) showed asymmetry of the thoracic cage with left lung volume reduction and mild mediastinal shift to the left. There were pleural thickening and encysted pleural effusion on the left, and atelectasis of adjacent lung portions. The right hemithorax showed small pleural effusion and thickening, in addition to a small nonspecific opacity in the right lung base. His heart was enlarged and showed pericardial calcifications.

His echocardiogram (Aug 17, 2005) showed septal and posterior wall thickness of 8 mm, and the following diameters: aorta, 25 mm; left atrium, 64 mm; diastolic left ventricle, 54 mm. His left ventricular ejection fraction was 40% (Simpson’s method), and there was diffuse hypokinesia. The right atrium and ventricle were very dilated, and the right ventricular systolic pressure was estimated at 42 mm Hg. The mitral and tricuspid valves showed moderate regurgitation, and the pericardium showed no change.

During hospitalization, the edema and the ascites subsided. The pleural effusion persisted, the right lower limb edema worsened, and scrotal edema developed.

His chest ultrasound (Sept 20, 2005) showed moderate multiseptated pleural effusion on the left side, with pleural thickening, basal atelectasis and immobility of the left dome of the diaphragm. There was no pleuro-pulmonary change on the right side, but the mobility of the right dome of the diaphragm was reduced. A new chest ultrasound (Sept 23, 2005) showed improved mobility of the diaphragm.

The pleural puncture showed a yellow fluid with total proteins of 0.3 g/dL and lactic dehydrogenase of 41 IU/L. The pleural biopsy revealed chronic pleuritis (Sept 23, 2005).

His lung tomographic angiography (Sept 25, 2005) evidenced pleural thickening, moderate pleural effusion and atelectasis of the left lung. On the right side, there was a small pleural effusion, including inside the lung fissure, and a 2.2-mm peripheral heterogeneous pulmonary nodule in the apical-posterior segment. Axillary and mediastinal lymph nodes were identified with diameters of up to 1 cm. The pulmonary trunk and branches showed no filling defect, but the pulmonary vascularization was reduced on the left side, due to compression resulting from the effusion and atelectasis.

The abdominal ultrasound (Sept 27, 2005) showed an enlarged liver and kidneys of preserved dimensions (right kidney, 9 cm, and left kidney, 8.5 cm).

The patient had fever (Sept 28, 2015), pulmonary infection was diagnosed, and vancomycin introduced.

The echocardiogram (Sept 28, 2005) showed mild and diffuse hypokinesia, septal and posterior wall thickness of 8 mm, and the following diameters: aorta, 28 mm; left atrium, 70 mm; diastolic left ventricle, 56 mm. The right atrium was markedly dilated, and the ventricle was dilated and moderately hypokinetic. The tricuspid valve was thickened, with impaired coaptation of its leaflets and severe regurgitation. There were indirect signs of pulmonary hypertension and moderate and diffuse impairment of the right ventricle.

Doppler ultrasound of the lower limbs (Sept 29, 2005) evidenced marked subcutaneous edema of the right lower limb, in addition to inguinal lymph nodes, and no sign of deep venous thrombosis.

The chest computed tomography (Sept 30, 2005) showed ground glass opacity in the right lung, compatible with edema, and left lung consolidation. There was left encysted pleural effusion with a hyperattenuating component inside, small area of encysted pneumothorax on the left side, mediastinal lymph nodes measuring up to 14 mm, and pericardial calcifications.

The chest ultrasound (Oct 3, 2005) showed a thick collection and consolidation in the left hemithorax.

The new chest computed tomography (Oct 7, 2005) showed no lymph node enlargement, a large pleural effusion with gas on the left side and pleural thickening, and hyperattenuating areas compatible with blood or purulent material. In addition, there were bilateral focal consolidation areas, more numerous on the right side.

The laboratory tests (Oct 13, 2005) revealed: hemoglobin, 8.8 g/dL; hematocrit, 29%; mean corpuscular volume, 91 µm^3^; leukocytes, 6300/mm^3^ (95% neutrophils, 2% lymphocytes, and 3% monocytes); platelets, 108,000/mm^3^; potassium, 3.3 mEq/L; sodium, 150 mEq/L; urea, 46 mg/dL; creatinine, 0.7 mg/dL.

The patient had diarrhea, and underwent colonoscopy (14 out 2005), which showed edematous mucosa with no inflammatory signs.

On October 16, 2009, the patient had septic shock, acute renal failure, requiring noradrenaline administration. Teicoplanin was introduced and hemodialysis performed. The shock became refractory and the patient died (Oct 18, 2005).

## Clinical aspects

The patient was a 28-year-old male with anasarca. There were moderate left systolic dysfunction and marked enlargement of the atria and right ventricle, with mild increase in pulmonary arterial pressure and moderate regurgitation of the atrioventricular valves.

Apparently diastolic dysfunction and dilatation of the ventricles and systolic dysfunction of the right ventricle predominated, indicating a restrictive heart disease.

Of the etiological possibilities, constrictive pericarditis and restrictive cardiomyopathies can be considered. The restrictive cardiomyopathies can be classified as non-infiltrative and infiltrative. Non-infiltrative restrictive cardiomyopathies are as follows: idiopathic cardiomyopathy, familial cardiomyopathy, hypertrophic cardiomyopathy, cardiomyopathy of systemic sclerosis, cardiomyopathy in pseudoxanthoma elasticum, and cardiomyopathy of diabetes. Infiltrative restrictive cardiomyopathies are present in the following diseases: amyloidosis, sarcoidosis, Gaucher disease, Hurler disease and fatty infiltration, and storage diseases (hemochromatosis, Fabry disease and glycogen storage diseases). The following endomyocardial diseases should be considered: endomyocardial fibrosis, hypereosinophilic syndrome, carcinoid heart disease, metastatic cancer, post-radiation, anthracycline cardiotoxicity, drugs causing fibrous endocarditis (serotonin, methysergide, ergotamine, mercurial agents, busulfan).

Of the restrictive cardiomyopathies, hypertrophic cardiomyopathy and amyloidosis are easily ruled out in our patient, because of the normal thickness of his left ventricular walls, and amyloidosis still for his lack of proteinuria, which is present in cases related to multiple myeloma.^1^

Hemochromatosis seems not to be the cause of this patient’s heart disease because his ferritin levels were not elevated, and his iron levels were low, although there was hyperkalemia, which might suggest hypoadrenalism, a condition present in hemochromatosis.

Sarcoidosis can be a cause of restrictive heart disease, but usually presents with ventricular arrhythmias, such as ventricular tachycardia and sudden death due to ventricular fibrillation, and intraventricular conduction block of the stimulus.^2^ Our patient had not a clinical course compatible with that diagnosis.

Regarding endomyocardial fibrosis, no apical amputation of the ventricles was observed on the echocardiogram, a pathognomonic finding of that disease.

Idiopathic restrictive cardiomyopathy could also be responsible for his clinical findings, because it has normal or reduced ventricular volumes and increased atria, with normal left ventricular thickness and normal or slightly altered systolic function. However, it is very rare, occurring more frequently in children younger than six years of age. It is caused by at least ten mutations in sarcomeric genes (troponins I and T, actin, myosin and titin), in addition to non-sarcomeric genes (desmin, laminin and transtirretin).^3-5^

Another cause of predominantly right heart failure would be chronic pulmonary thromboembolism, but the tomographic angiography of his pulmonary arteries showed no abnormality.

Finally, the patient’s clinical findings could result from constrictive pericarditis.

Despite the presence of anasarca, dilatation of the atria and biventricular systolic dysfunction, his BNP levels were only slightly elevated (192 pg/mL), while the expected BNP levels for that pathology would exceed 500 pg/mL.^6^ However, Fernandes et al. have found low levels of BNP in a recently published series of pericarditis.^7^ The same has been evidenced by Reddy et al., who have reported a mean BNP level of 116 pg/mL in pericarditis, and of 726 pg/mL in restrictive cardiomyopathy.^8^

The predominance of pleural effusion on the left side, rather than on the right side, is commonly seen in heart failure. In addition, left pleural effusion has been associated with cases of constrictive pericarditis.^9,10^

Although the echocardiogram showed no pericardial involvement, because there was neither pericardial effusion nor thickening, constrictive pericarditis cannot be ruled out, because that test is little sensitive to reveal pericardial thickening in the absence of effusion. Oh et al. have reported pericardial thickening in only 36% of the cases published.^11^ Doppler-echocardiographic findings significantly increase the method’s sensitivity.^11^

Magnetic resonance imaging would have been useful, because it defines precisely the pericardial thickness, which is better seen during the systole and measures 2 to 4 mm.^12,13^ By use of gadolinium, magnetic resonance imaging can show either the uniform and smooth thickening compatible with acute or subacute pericarditis or the irregular thickening of chronic constrictive pericarditis, pericardial fibrosis, tumors or metastases. The visualization of the pericardium depends on the presence, amount and extension of the subepicardial fat. Magnetic resonance imaging can outline completely the pericardium on the right ventricle, but only 60% of it on the lateral wall of the left ventricle.^14^

Although the echocardiogram of our patient showed no pericardial abnormality, his chest tomography evidenced pericardial calcifications that could be attributed to chronic constrictive pericarditis.

Thus, favoring chronic constrictive pericarditis, we have pericardial calcifications, low BNP levels, and pulmonary arterial pressure levels below 50 mmHg.

Regarding etiology, the constrictive pericarditis could be idiopathic (probably viral) or due to tuberculosis.

Chronic liver diseases, due to both viral hepatitis and other causes of cirrhosis, can progress with pulmonary hypertension. Ramsey et al.^13^ have reported pulmonary hypertension in 10% of the candidates for liver transplantation. However, our patient had negative serologies for viral hepatitis, and autoimmune hepatitis was ruled out because of the absence of antinuclear antibodies, and anti-smooth muscle and anti-microsomal antibodies.^13^ (Desiderio Favarato, MD)

**Diagnostic hypothesis**: restrictive syndrome due to constrictive pericarditis; final event: septic shock. (Desiderio Favarato, MD)

## Postmortem examination

**Figure 2:**
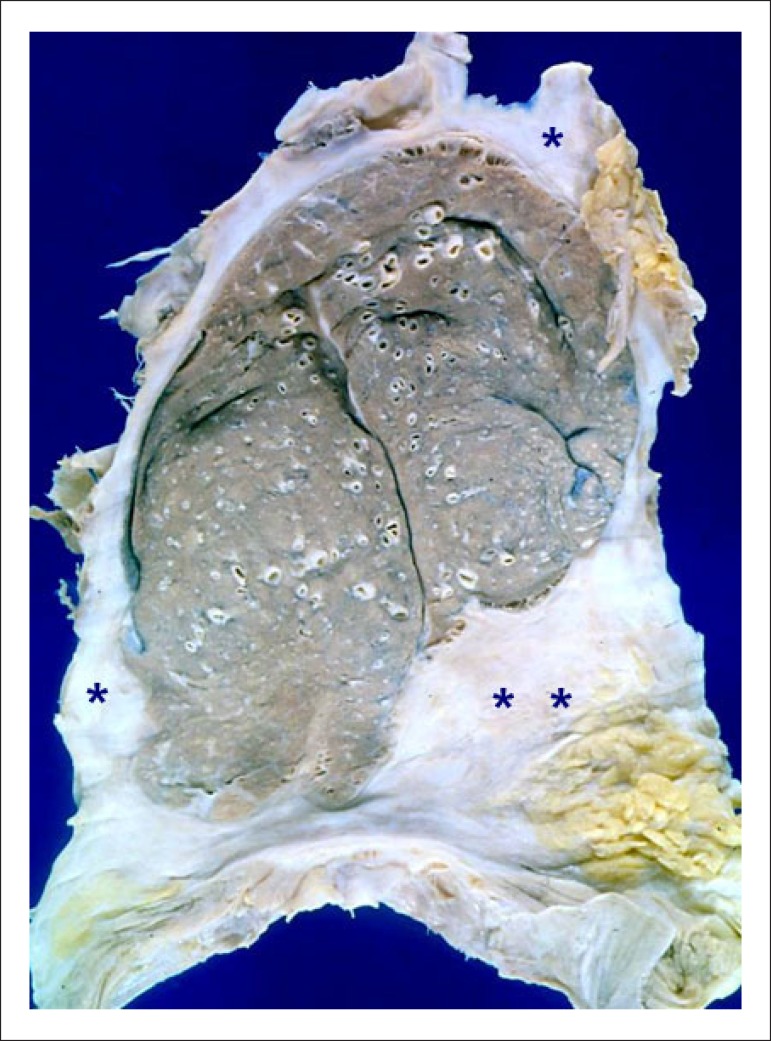
Section of the left lung showing chronic, obliterative pleural fibrosis (asterisks).

**Figure 3:**
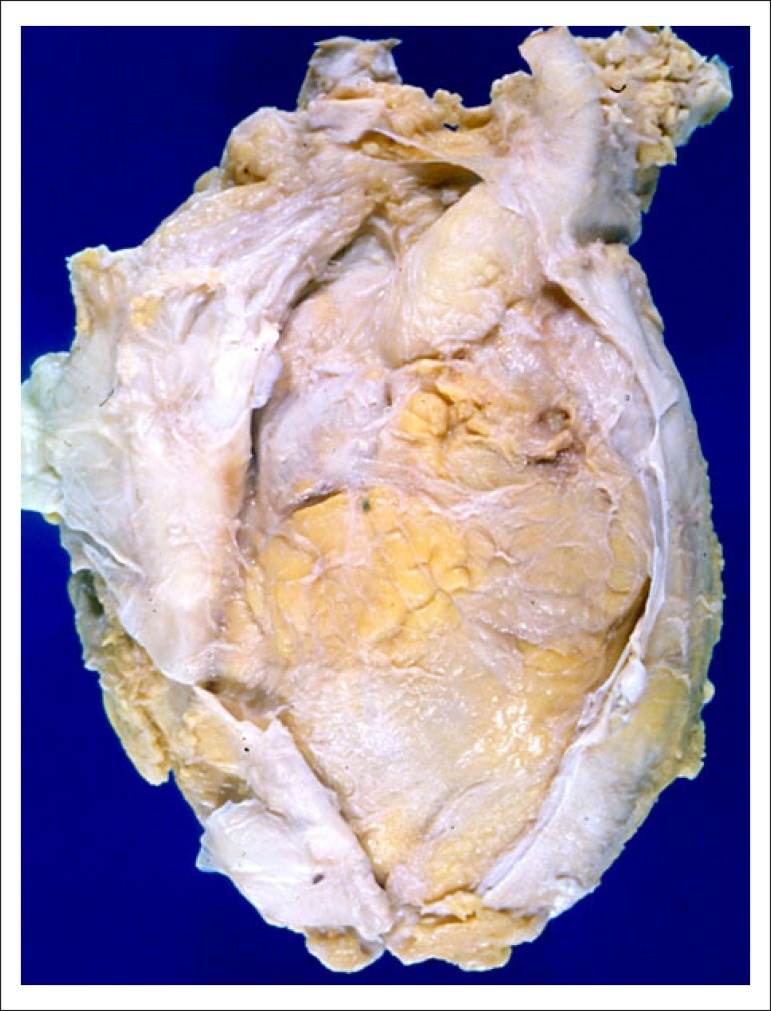
Anterior view of the heart with the pericardial sac opened, evidencing the whitish thickening of the pericardium and epicardium, which are adhered, characterizing constrictive pericarditis.

**Figure 4:**
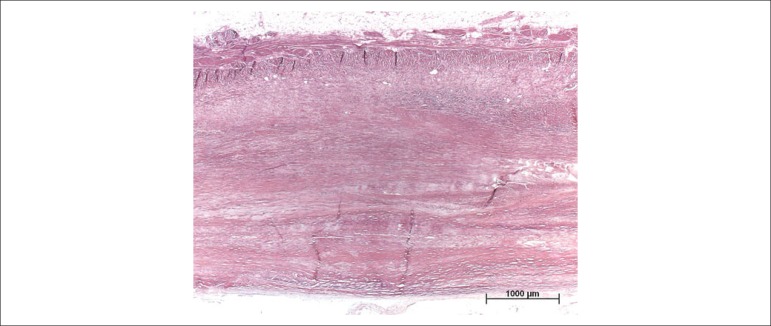
Histological section of the pericardium evidencing marked thickening due to dense fibrosis; absence of inflammatory process or of granuloma (Hematoxylin-Eosin).

**Figure 5:**
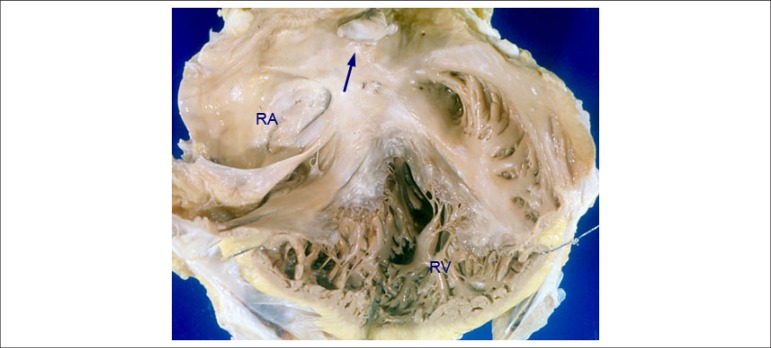
Opened heart showing the right chambers. Note the marked dilatation of the right atrium (RA), with a small thrombus attached to the endocardium (arrow). The right ventricle (RV) shows mild dilatation and normal wall thickness, and dilatation of the tricuspid valve ring.

After opening the chest wall, strong adherence of both lungs to the rib cage and diaphragm was observed, more exuberant on the left side. The parietal and visceral pleurae were fused, whitish and markedly thickened, and incarceration and atelectasis of the left lung were seen (Figure 2). The pericardium was whitish and thickened, firmly adhered to the epicardium, with calcifications on the antero-superior region (Figure 3). The histological examination of the pleurae and pericardium evidenced dense fibrosis with areas of hyalinization, neovascularization and isolated foci of mild mononuclear inflammatory infiltrates; there was no granuloma (Figure 4). The heart weighed 472 g. Both atria were markedly dilated, with a small organizing thrombus attached to the right atrial endocardium. The atrioventricular valve ring was wide, mainly the right one (Figure 5). The right ventricle was mildly dilated, and the left ventricle was normal. The histological examination of the myocardium evidenced no abnormality. The inferior vena cava was dilated, with thrombi attached to the endothelium. The pulmonary parenchyma showed chronic passive congestion with histological evidence of pulmonary hypertension, organizing diffuse alveolar damage and foci of bronchopneumonia on the right lung. The abdominal cavity showed signs of chronic ascites, with peritoneal fibrous thickening and intestinal adhesions. The liver and the spleen showed marked chronic passive congestion, and the spleen also had extensive areas of recent infarction. In addition, the kidneys showed acute tubular necrosis, and the liver, foci of hemorrhagic necrosis in the centrilobular region. (Luiz Alberto Benvenuti, MD).

**Anatomopathological diagnoses** - chronic constrictive pleuropericarditis; mixed hemodynamic shock (cardiogenic and septic); focal bronchopneumonia in the right lung. (Luiz Alberto Benvenuti, MD)

## Comments

The patient was a 28-year-old male with heart failure, and pleural effusion and thickening. The postmortem examination showed chronic constrictive pericarditis associated with intense pleural fibrosis and lung incarceration on the left side, characterizing chronic pleuropericarditis. That condition results from the resolution of the pleural and pericardial inflammation, which can have several etiologies, such as tuberculosis, collagen diseases, side effect of drugs or radiotherapy, uremia, inflammatory bowel disease, viral infection, and chest traumas or surgeries.^15^ In addition, the more recently characterized IgG4-related disease can be a cause of chronic pleuropericarditis.^16^ Classically related to tuberculosis, the constrictive pericarditis etiology in developed countries has changed, with an increase in the idiopathic etiology or that following surgery or radiotherapy. In developing countries, however, tuberculosis remains the most common etiology.^15,17^ In the present case, there was neither chronic kidney disease nor a clinical history compatible with the secondary effect of drugs or radiotherapy. There was no clinical evidence of inflammatory bowel disease or collagen disease, and the histological examination of the pleurae and pericardium showed no inflammatory infiltrate rich in plasma cells that could suggest the occurrence of IgG4-related disease; the lesions were essentially fibrotic, with a very mild inflammatory infiltrate. Tuberculosis should be considered, because of its high prevalence among us, but no direct evidence of it, such as granuloma or its remnants, was found. Thus, we concluded that our patient had a chronic idiopathic pleuropericarditis, because its etiology is not clear. In such cases, the viral etiology is a possibility, in addition to tuberculosis itself. The terminal cause of death was mixed hemodynamic shock (cardiogenic and septic), confirmed by the presence of focal necrosis in multiple organs (centrilobular region of the liver, renal tubules and spleen) and areas of bronchopneumonia. (Luiz Alberto Benvenuti, MD).

**Section editor:** Alfredo José Mansur (ajmansur@incor.usp.br)

**Associated editors:** Desidério Favarato (dclfavarato@incor.usp.br)

Vera Demarchi Aiello (vera.aiello@incor.usp.br)
